# Diversity of Alticinae in Oaxaca, Mexico: A preliminary study (Coleoptera, Chrysomelidae)^1^

**DOI:** 10.3897/zookeys.332.4790

**Published:** 2013-09-19

**Authors:** David G. Furth

**Affiliations:** 1 Department of Entomology, National Museum of Natural History, Smithsonian Institution, Washington, D. C. 20560

**Keywords:** Mexico, Oaxaca, diversity, Coleoptera, Chrysomelidae, Alticinae, endemism, fieldwork, collections

## Abstract

This is a preliminary study of the diversity of the Flea Beetles (Alticinae) of the Mexican state of Oaxaca based on fieldwork by the author in 1991, 1997, and 2010, the literature, and specimens in several institutional collections. The number of genera and species for Mexico as well as for Oaxaca increased significantly from previous studies. There are now 625 species in 90 genera recorded from Mexico with 275 species in 68 genera recorded from Oaxaca. There are 113 species known only from the state of Oaxaca and another 38 species known only from Oaxaca and the surrounding states. Oaxaca has a relatively high diversity as well as a high percentage of endemism. This study also demonstrates the effects of how even a small amount of fieldwork together with extracting specimen data from institutional collections can significantly increase the total faunistic and diversity knowledge of an area. A complete list of the genera and species known from Oaxaca is included.

## Abstract (es)

Este es un estudio preliminar de la diversidad de los Escarabajos Pulga (Alticinae) del estado mexicano de Oaxaca basado en el trabajo de campo por el autor en 1991, 1997, y 2010, la revisión de la literatura y el estudio de varios especímenes en colecciones institucionales. El número de géneros y especies para México y para Oaxaca aumentó significativamente a partir de estudios anteriores. En la actualidad hay 625 especies en 90 géneros registrados para México con 275 especies en 68 géneros registrados de Oaxaca. Hay 113 especies conocidas sólo del estado de Oaxaca, y otras 38 especies conocidas sólo de Oaxaca y los estados circundantes. Oaxaca tiene una diversidad relativamente alta, así como un alto porcentaje de endemismo. Este estudio también demuestra como una pequeña cantidad de trabajo de campo junto con la extracción de datos de especímenes de colecciones institucionales pueden aumentar significativamente el conocimiento sobre la fauna total y la diversidad biológica de una región. Se incluye una lista completa de los géneros y especies conocidas de Oaxaca.

## Introduction

Although Mexico is the fourteenth largest country in the world (ca. 2,000,000 km^2^) it is the fifth most biodiverse country and is one of the 25 biodiversity hotspots (Mittermeier 1988; Mittermeier et al. 1999).

**Map 1.**
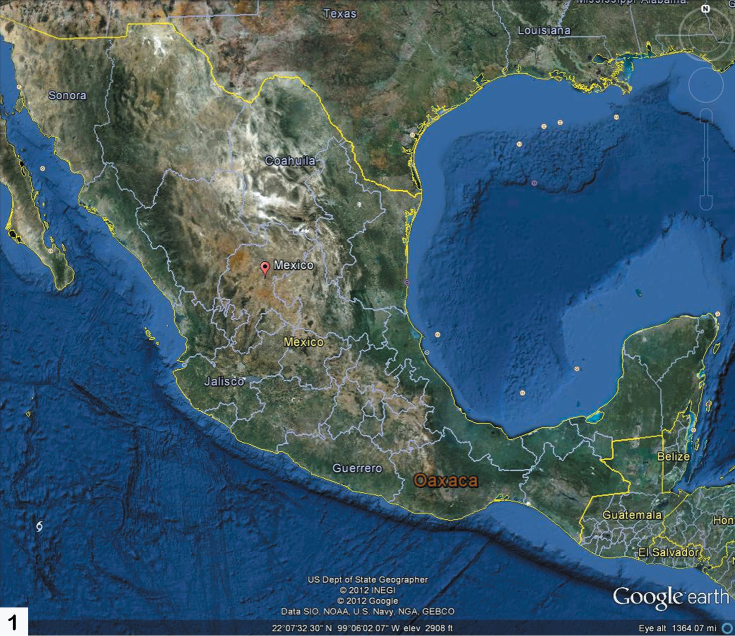
Mexico and southern USA, depicting the position of the state of Oaxaca.

**Map 2.**
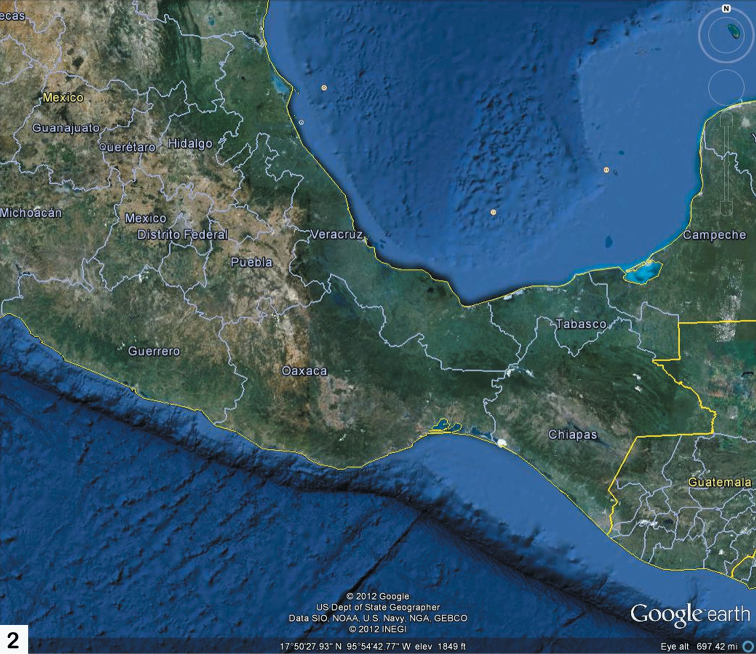
Oaxaca with the surrounding states and demonstrating the strong geographical constriction of the Isthmus of Tehuantepec.

**Map 3.**
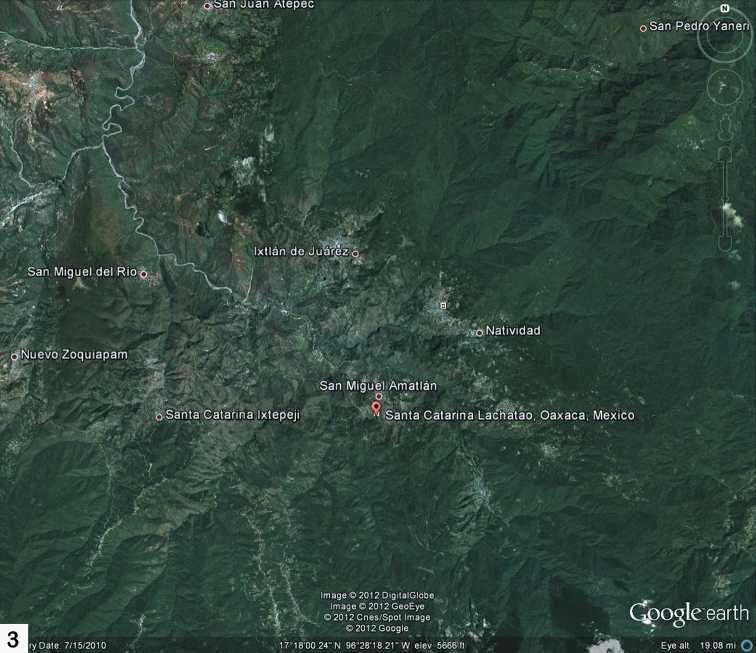
Google Earth view of the Sierra de Juárez mountains with the current study’s home base of Ixtlán de Juárez and some of the collecting localities from the 2010 field trip, especially Santa Catarina Lachatao.

**Map 4.**
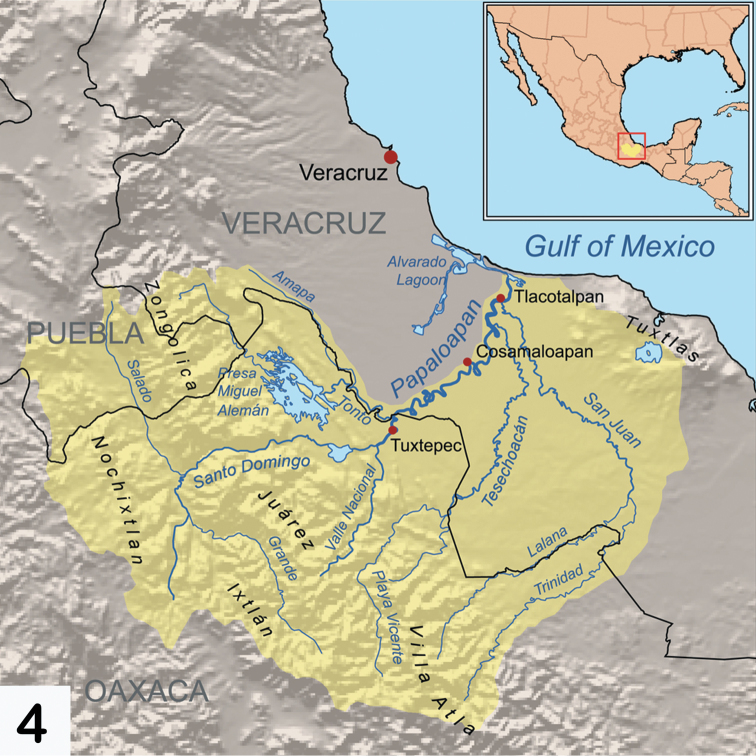
The Sierra de Juárez mountains of Oaxaca with the surrounding smaller mountain systems.

Oaxaca is one of the most mountainous and rugged areas in Mexico and it is geologically complex as well (Ferrusquía-Villafranca 1993). Its southern and central areas are composed of the Sierra Madre del Sur mountain range, one of the major ranges in Mexico. However, the mountains of Oaxaca are actually composed of several less extensive ranges. The primary one is the Sierra Madre de Oaxacathat is a mountain range just north of Sierra Madre de Sur, but converging with it. It begins in the state of Veracruz at Pico de Orizaba and extends in a southeasterly direction for 300 km until reaching the Isthmus of Tehuantepec. Mountain peaks in the Sierra Madre de Oaxaca average 2,500 m in elevation, with some peaks exceeding 3,000 m. The Sierra Madre de Oaxaca also can be split into many smaller sierras, each with unique environments and human inhabitants, including Sierra de Juárez (this study area) and Sierra Mazateca (to the northwest) (Maps 1, 2). Of special interest to this study is its home base in Ixtlán de Juárez, a mountain community for the environmentally aware. Here, locals have developed a special eco-tourism project where guests are taken on guided tours through the area’s attractive forests. Within the same hour, you can experience a hot, dry climate, and then ascend the mountains to a cold damp region (Map 3). The Sierra de Juárez is a range of mountains in Oaxaca state, Mexico between latitudes 17°20'–17°50'N and longitudes 96°15'–97°00'W, with an area of about 1,700 km². The range is separated from the Sierra de Zongólica to the north by the Santo Domingo River, flowing through the Tecomavaca Canyon. It stretches south-eastward to the Cajones River and the Sierra de Villa Alta. The mountains are in the district of Ixtlán de Juárez in the Sierra Norte de Oaxaca region. It is named after Mexico’s only indigenous president, Benito Juárez, who was born here in 1806 in the small village of San Pablo Guelatao (Map 4). These mountains climb from 500 m to 3,250 m, with many large and deep ravines. They are formed of folded sedimentary rocks with series of younger granitic intrusions that date from the Palaeozoic to Cenozoic, with the majority being Mesozoic. The climate is subtropical in the lower regions and temperate and subhumid above 1000 m, with average temperature from 16°–20° C. There is regular frost in the higher mountains. Annual rainfall, fed by the trade winds from the Caribbean Sea, ranges from 700 mm to 4000 mm or more. The Valle Nacional River originates in the Sierra de Juárez, one of the major tributaries of the Papaloapan River (Map 4).

The Sierra de Juárez is one of Oaxaca State’s wettest areas and richest in forest diversity, with perhaps 2000 of the 8000 or more plant species that are found in the state. It is mostly covered by montane cloud forest, but includes tropical evergreen forests and forests of pine, pine-oak and oak. The cloud forest forms a band from 1,000–1,400 m in height up to 2,250 meters along the northern and eastern slopes. The cloud forest climate is cool (14°–20° C) and has mean annual rainfall that exceeds 2,000 mm and is sometimes much higher. The dominant trees are 20–30 m tall and include evergreen and deciduous species, palms, tree ferns, heather shrubs, vines, and moisture-loving herbs (the facts above were taken from Wikipedia).

As evident from Maps 1 and 2 Oaxaca is positioned in a rather unique biogeographical part of Mexico and, in fact, Central America. It is bordered north and west by the rather dry and deep Rio Balsas (an almost west-east) transect of the state of Guerrero. To the north and east is the southern-most aspect of one of the other major mountain ranges of Mexico, the Sierra Madre Oriental, at that point in Veracruz. The eastern most part of Oaxaca spans the Isthmus of Tehuantepec that borders the state of Chiapas. The Isthmus of Tehuantepec is not mountainous and is distinctly the narrowest part of Mexico where the states of Oaxaca, Veracruz, Chiapas and Tabasco converge. This geographical constriction certainly has an effect on the diversity and distribution of the fauna and flora. Presumably to the south the biodiversity is predominantly Neotropical.

For Mexico’s phanerogamic flora the highest diversity is found along a belt originating in Chiapas, traversing Oaxaca, and continuing to central Veracruz in the east and to Sinaloa and Durango on the west and cloud and evergreen forests are the most diverse per unit area, endemism is prevalent, and Oaxaca has a higher number of species than any state (Rzedowski 1993).

Llorente-Bousquets et al. (1993) report that based on butterflies (Papilionoidea) the two most species-rich areas in Mexico are the Sierra de Juárez (the area of the present study) and Los Tuxtlas (Veracruz), with the highest numbers of species in Oaxaca (40), Chiapas (41), and Veracruz (41). In a survey of 20 different groups of arthropods (8,599 species), the most diverse states were Veracruz (2072), Chiapas (1306), Oaxaca (1256), Guerrero (1124) (Llorente-Bousquets et al. 1996).

The current study is part of a series of publications on the diversity of Alticinae (Flea Beetles) in Mexico (Furth 1998, 2004, 2006, 2007, 2009; Savini et al. 2001). Besides elucidating the biological diversity of Mexico based on this taxon of herbivores, it provides an example of how the historical literature, historical collecting based on specimens in institutional collections, and new fieldwork can be combined to relatively rapidly assess such diversity. Although the historical literature is a fixed entity, when more institutional collections are examined or surveyed for historical collecting records and when more targeted fieldwork is conducted (even for short periods), there is a significant increase in diversity knowledge very quickly.

Furth and Savini (1996, 1998) listed all Alticinae known from Central America with their known distribution. Furth (2004) published the first accounting of Alticinae diversity in Mexico based primarily on the historic literature as well as some specimens from collections at the USNM, a few borrowed specimens from other collections, and some from very brief collecting by the author in 1991, 1993, 1995, and 1997. At that time there were 501 species in 85 genera listed from Mexico of which 96 species in 33 genera were recorded from Oaxaca. This made Oaxaca the third most diversity state behind Veracruz (182 species) and Guerrero (124 species), and just ahead of Durango (87 species) and Tabasco (74 species). Because most of these data were based on very old and sporadic collecting, they were very preliminary and also may have reflected the accessibility or popularity of certain locations. Nevertheless, the biological diversity was understandably higher in most of the southern Mexican states, e.g., Chiapas, Guererro, Oaxaca, Tabasco, and Veracruz presumably due to their proximity to the Neotropics. This has also been documented for other groups of animals, including insects, such as for Odonata (González and Novelo 1996), Psocoptera (Mockford and Aldrete 1996), Passalidae (Reyes-Castillo 2002), and Bruchinae (Nápoles 2002).

In Furth (2006) new data was added that changed the Alticinae diversity in the various Mexican states, but Oaxaca remained third most diverse with 122 species, Guerrero remained second with 141, and Veracruz was the most diverse with 198. Other states with significant diversity (Furth 2006) were Durango (97 species), Morelos (84), Tabasco (81), and Chiapas (81). It is noteworthy that of these seven most diverse states, five are surrounding Oaxaca.

As discussed in Furth (2004) in Mexico there is a major biogeographic transition zone between the Nearctic and Neotropical Regions and biogeographic affinities may also vary greatly depending on the group considered. Also levels of endemism vary greatly depending on the group considered and, of course, depending on the relative knowledge of the group. As with any country some vertebrate and plant groups are well known, whereas most insect groups are not. Aspects of Mexican biogeography and endemism were also discussed in Furth (2004) with some examples from other groups provided. Biogeography and endemism will be treated below relative to the data from this study regarding Oaxaca and surrounding states.

## Materials and methods

The data for this study was taken from three primary sources: first, from previously published literature, especially Furth (2004), Furth (2006) that included original published literature, including Furth and Savini (1996); second, from museum specimens borrowed from a variety of collections as follows: University of California, Berkeley (UCB); University of California, Davis (UCD), California Academy of Sciences (CAS); California Department of Food and Agriculture (CDFA); Brigham Young University (BYU); American Museum of Natural History (AMNH); U. S. National Museum of Natural History (USNM); Texas A. & M. University (TAMU); The Natural History Museum, London (NHM); the F. C. Bowditch Collection, Museum of Comparative Zoology, Harvard University (MCZ); Naturhistorisches Museum Basel (Switzerland) (NHMB); Zoologisches Staatssammlungen München (Germany) (ZSMC); third, from fieldwork by the author in 1991 (19-23 August) around Oaxaca City and in the Sierra de Juárez, 1997 (22-23 July) around Oaxaca City and southwest along Route 190, and 2010 (29 July-4 August) around Oaxaca city and in the Sierra de Juárez.

The Appendix is a combination of older records from the literature, a few collections (USNM, MCZ, NHMB), and new collection records from 8 other institutions above and the author’s field work (DGF 1991, 1997, 2010).

Examination and determination of the specimens was made using a Leica MZ APO binocular dissecting microscope. The digital photos of Figure 10 were produced by Karolyn Darrow using the Visionary Digital^TM^ imaging system and Adobe Photoshop^TM^.

The fieldwork was primarily based out of the Universidad de la Sierra Juárez (USJI) (Figure 11). Alticinae were collected by general and/or host plant-targeted sweeping with a 15 inch diameter aerial insect net using an aspirator. The majority of the field sites were in the vicinity of Santa Catarina Lachatao (SCL) and daily trips were accompanied by Prof. Atilano Contreras Ramos (UNAM), Prof. Jose Arturo Casasola (USJI), and various members of the SCL community (Figure 12). After the fieldwork extensive collection examination and curation was done at the Colección Nacional de Insectos, Instituto de Biología, UNAM (Figure 13, 14)

## Results

**Figure 1.**
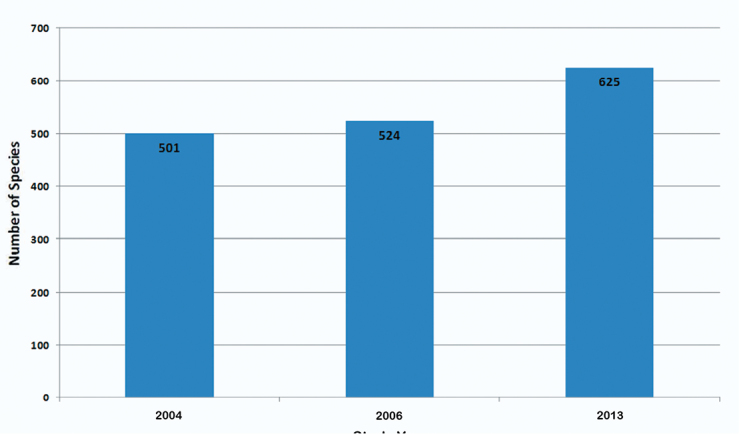
The total number of Alticinae species recorded from Mexico from Furth (2004, 2006) and the current study.

**Figure 2.**
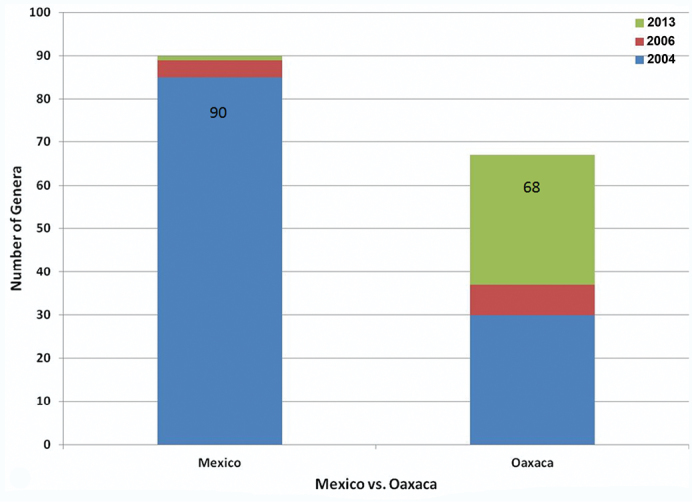
The total number of genera recorded from Mexico and Oaxaca based on Furth (2004, 2006) and the current study.

**Figure 3.**
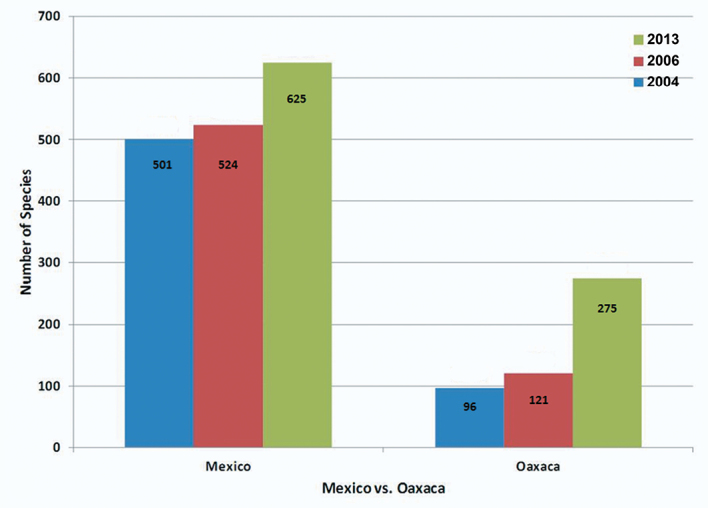
The changes in number of species recorded from Mexico versus Oaxaca only, based on Furth (2004, 2006) and the current study.

**Figure 4.**
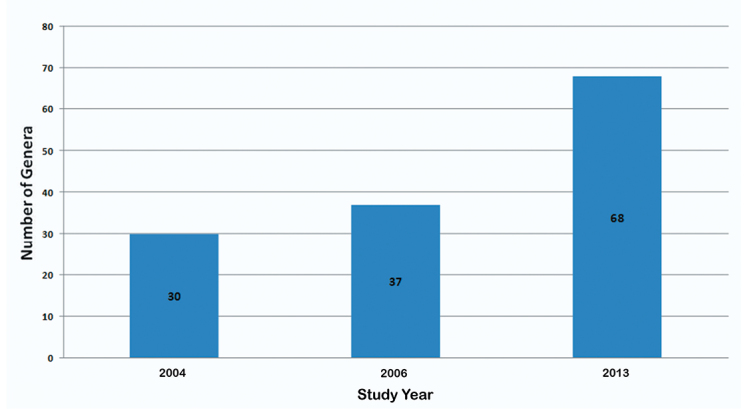
The changes in number of genera of Alticinae recorded only from Oaxaca from Furth (2004, 2006) and the current study.

As a result of a week of fieldwork in Sierra de Juárez of Oaxaca, Mexico, in 2010 and subsequent determination of the specimens collected, as well as examination of several institutional collections, the number of known species of Alticinae of Mexico increased from 524 (Furth 2006) to 625 (Figure 1) - an increase of over 19%. At the generic level there was only one genus added to the overall fauna of the country (Figure 2). Also resulting from the new fieldwork and collections examined, the number of recorded species for the state of Oaxaca increased from 121 (Furth 2006, 122 were reported but one found later to be in error) to 275 (Figure 3) – an increase of almost 79% and the number of Oaxacan genera rose from 37 to 68 (Figure 4) – an increase of 84%. At both the species and generic levels in Oaxaca these increases were significantly more than the increases from Furth (2004) to Furth (2006) for species of 96 to 121 (26%) (Figure 3) and for genera from 30 to 37 (23%) (Figure 4).

**Figure 5.**
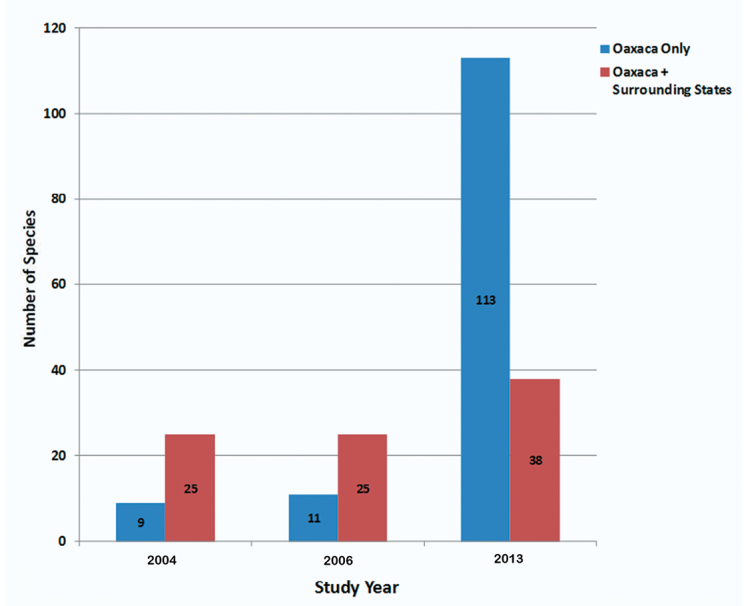
A comparison of the number of endemic species of Alticinae from Oaxaca only and from Oaxaca plus the surrounding states as recorded in Furth (2004, 2006) and the current study.

**Figure 6.**
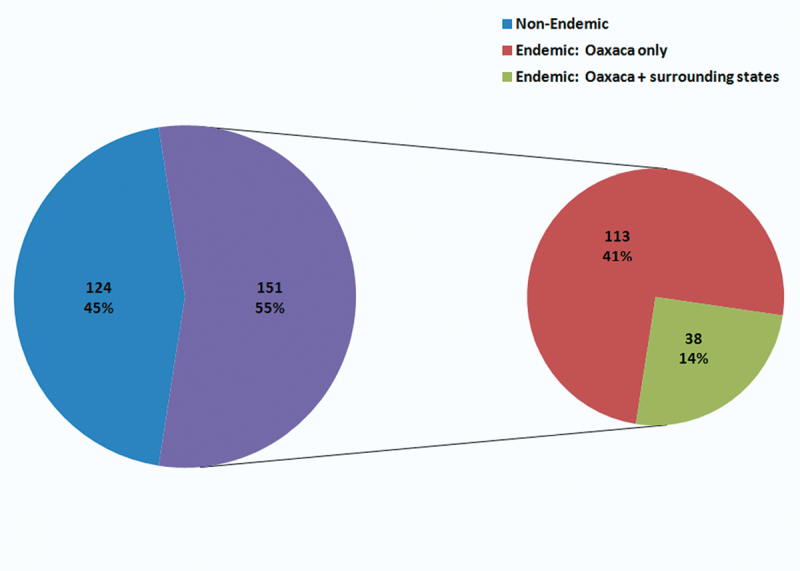
Endemic and non-endemic species numbers and percentages as recorded from the current study.

As for the endemism of Oaxaca as demonstrated by the Alticinae, Figure 5 shows that in Furth (2004, 2006) there were 9 and 11 species, respectively, recorded only from the state of Oaxaca, but as a result of the 2010 fieldwork there are 113 species – an increase of almost 930% from Furth (2006). Many of these (81 species or 72 %) currently only have morphospecies names and probably a significant proportion of these are new to science (see Appendix for OM species numbers). If the endemism is examined at a somewhat broader perspective, i.e., including species recorded in Oaxaca as well as the surrounding states (those bordering Oaxaca, plus Tabasco) then the endemic species from the 2010 data is a less dramatic increase from Furth (2004, 2006) or 25 to 38 – an increase of 52% (Figure 5). This means that of the 275 species recorded from the 2010 fieldwork from Oaxaca, 155 (55%) species are endemic at some level; 41% are restricted endemics known only from Oaxaca and 14% are more broadly endemic; known also from surrounding states (Figure 6).

**Figure 7.**
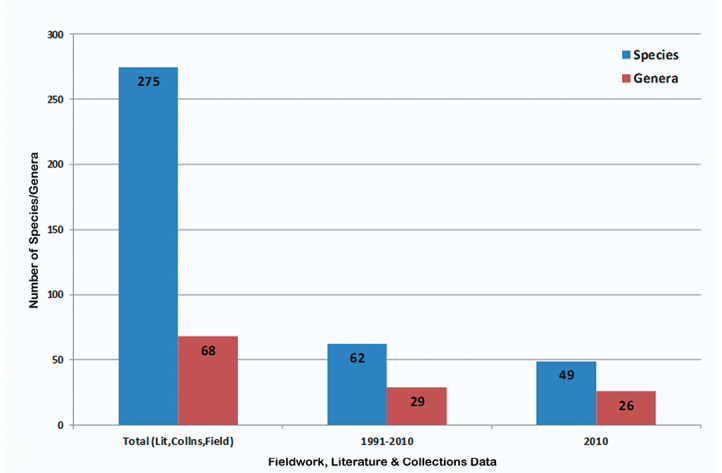
A comparison of the numbers of species/genera recorded from total evidence (literature, collections, author fieldwork), from all fieldwork (1991, 1997, 2010), and from the 2010 field trip alone.

Figure 7 shows the numbers of species (62) and genera (29) collected by the author during fieldwork from different trips to Oaxaca (1991, 1997, 2010). Of these the 2010 collecting trip alone resulted in 49 species and 26 genera. The 1991 collecting trip was 5 days, the 1997 trip 2 days, and the 2010 trip 7 days. Thus, the 2010 trip alone produced 79% of the species and 90% of the genera (Figure 7).

**Figure 8.**
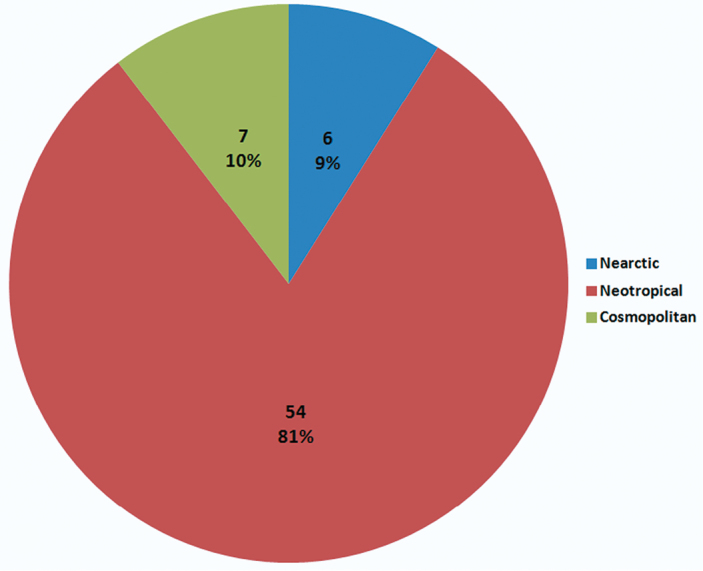
The biogeographic affinity of Alticinae genera of Oaxaca. Cosmopolitan genera are those found in several biogeographic regions.

Examining the biogeographical affinities of the Alticinae diversity of Oaxaca at the generic level, i. e, the biogeographic affinities of the 68 genera recorded, there are 6 (9%) genera of Nearctic affinity, 7 (almost 10%) of Cosmopolitan affinity, and 54 (81%) of Neotropical affinity (Figure 8).

**Figure 9.**
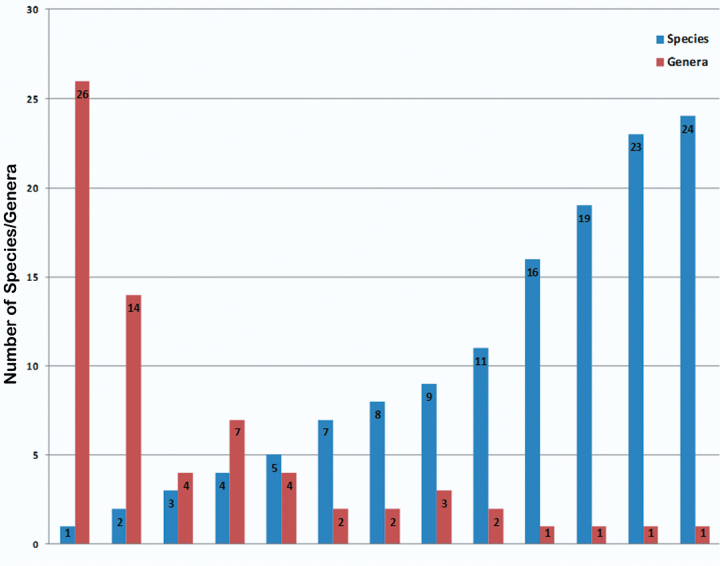
The number of species per genus of recorded Oaxacan Alticinae.

Another way to look at the diversity of the Oaxacan Alticinae is to examine the number of species per genus. As shown in Figure 9 of the 68 genera recorded from Oaxaca there is a high number of genera (26) with only one species and a high number of species (16 + 19 + 23 + 24) or 82 from only one genus, with a trend towards more species from fewer genera.

**Figure 10.**
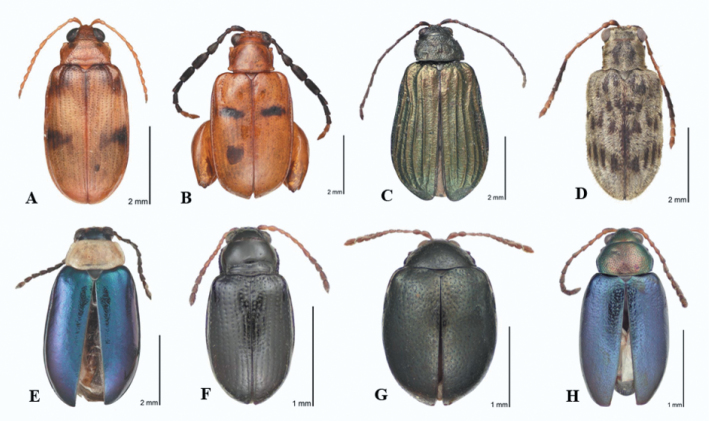
Examples of newly recorded Alticinae from the current study: **A** *Sphaeronychus* OM sp. 2 **B** *Allochroma* OM sp. 1 **C** *Deuteraltica* OM sp.1 **D** *Hypolampsis* OM sp. 2 **E** *Disonycha nigrita* **F** *Trichaltica zapotensis* **G** New Genus **H** *Phyllotreta aeneipennis*.

Figure 10 illustrates a few representatives of Alticinae genera and species that demonstrate presumed endemism and significant affinities of the biogeographical elements and distributional extensions in Oaxaca. *Sphaeronychus* OM sp. 2. (Figure 10A) represents one of two probable new species in a genus known from Brazil (25 species), one each from Ecuador and Peru, and only 2 known species from Central America. *Allochroma* OM sp. 1 (Figure 10B) is a probably new species representing a Neotropical genus with 11 known species from Mexico (Furth 2006), another 18 from Central America, and about 7 from South America. *Deuteraltica* OM sp. 1 (Figure 10C) is an undescribed species of a monotypic genus only known from Mexico, Guatemala, and El Salvador (Furth and Savini 1996). *Hypolampsis* OM sp. 2 (Figure 10D) is a probably new species of a very large genus (possibly the largest Neotropical genus of Alticinae) with only 4 known species from Mexico (Furth 2006), another 15 known from elsewhere in Central America (Furth and Savini 1996), and more than 60 from South America. *Disonycha nigrita* Jacoby (Figure 10E) is new to Mexico from the south, previously known only from Guatemala and El Salvador. *Trichaltica zapotensis* (Jacoby) (Figure 10F) is new to Mexico from the south, only previously only known from Guatemala and originally described as a species of *Crepidodera*. New Genus OM A (Figure 10G) is almost certainly a new genus probably of Neotropical affinity. *Phyllotreta aeneicollis* Crotch (Figure 10H) is a Nearctic element, new to Mexico from the north, previously only known from southeastern, south central, southwestern USA.

**Figure 11.**
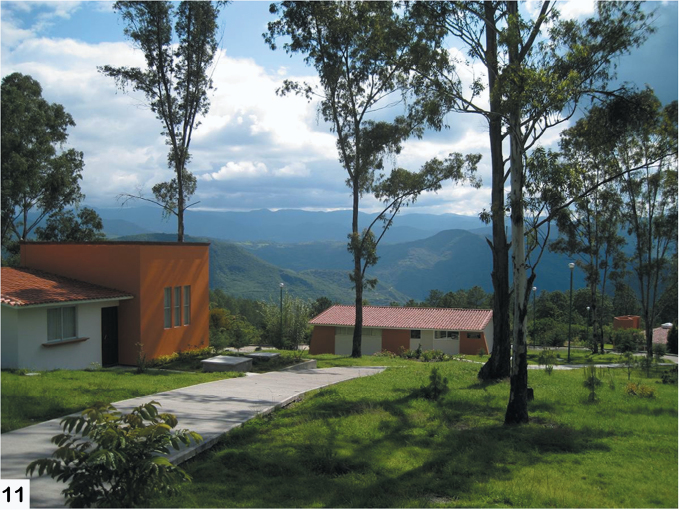
View of the cloud forest environment of Sierra de Juárez mountains from the Universidad de la Sierra Juárez campus.

**Figure 12.**
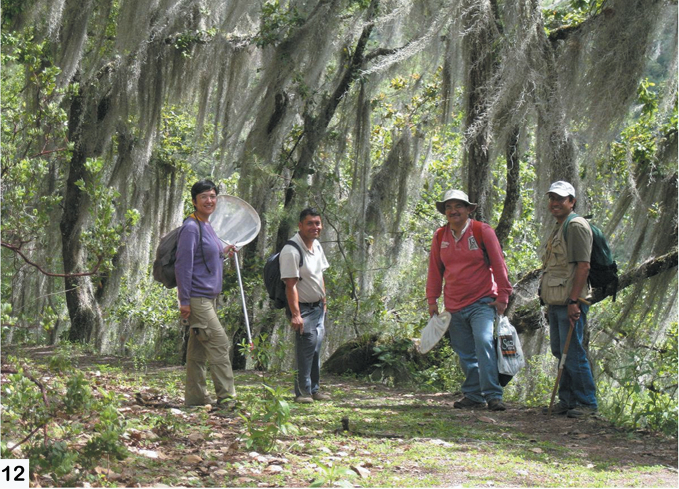
A view of typical Bromeliad-dominant cloud forest (many trees covered by *Tillandsia usneoides* (Linnaeus) (L.) Bromeliaceae) around Santa Catarina Lachatao with some of the 2010 collecting team (right to left: Jose Arturo Casasola, Atilano Contreras-Ramos, a local guide, Diana X. Munn).

**Figure 13.**
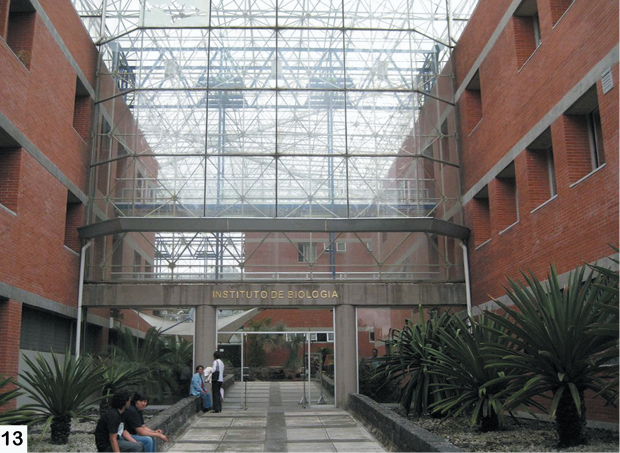
Entrance to the Instituto de Biología (UNAM) where the Mexican National Insect Collection is housed.

**Figure 14.**
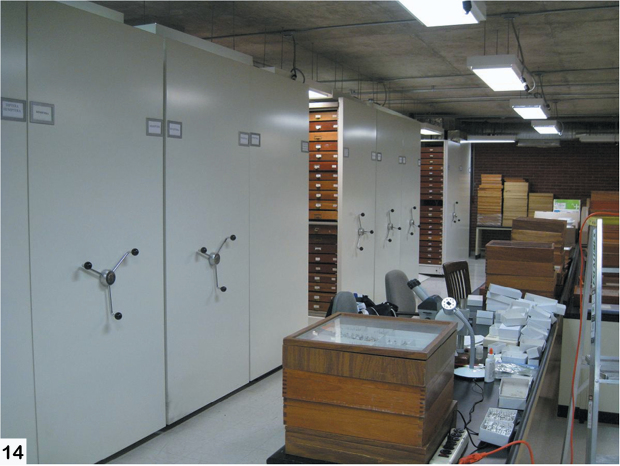
Compactors of the Mexican National Collection (UNAM, IB).

## Discussion

As indicated in the Introduction and evident from Maps 1–4, Mexico is geographically positioned rather uniquely between North America and South America and with a large diversity of landscapes, climates, and microhabitats; these are reflected in its diversity of flora and fauna. The southern state of Oaxaca is an interesting reflection of this diverse Mexican geography and its habitats with its own set of special features.

The data in Furth (2004, 2006) were compiled primarily from researching the historical and more recent literature as well as from searching and determination of a few collections (i. e., the USNM, MCZ, and NHMB). The author’s previous studies of Alticinae diversity of Mexico were published (2004, 2006) and were based on the literature and examination of primarily two research collections. The current study resulted from more extensive examination of collections from a variety of institutional research collections and a single, brief field trip to one area of Oaxaca. This multi-faceted strategy of reviewing the literature, then searching and examining historical research collections at a larger variety of institutions, as well as increased fieldwork is demonstrated well by the current study. Primarily as a result of the rather brief 2010 fieldwork coupled with the study of at least eight additional institutional collections the number of species known from all of Mexico as well as from Oaxaca increased dramatically, 19% and 79%, respectively. After all examination of historical collections or “indoor collecting”, as it is sometimes called, is the result of many different collecting events (and methods) over many years by different collectors. Also targeted fieldwork by an expert produces significant increase in the known fauna in a relatively short time. The efficacy of the combination of these two aspects (examining new collections and new collecting) is demonstrated by the significant increase in Oaxacan Alticinae diversity by 79% for species and 84% for genera.

The three expert field collecting trips by the author in 1991, 1997, and 2010 were of different lengths and, in the case of 1991, at somewhat different seasons. In each case at least one day was spent collecting in the general vicinity of Oaxaca City, but the 1991 and 2010 field trips overlapped considerably geographically. Therefore, the increase in recorded Alticinae diversity for Oaxaca is due to the addition of a significant number of institutional collections examined as well as the intensive 7 days of fieldwork in 2010.

As mentioned above in the Introduction several previous studies of various members of the flora and fauna have demonstrated the high levels of endemism in southern Mexico, especially in Oaxaca. Again, the current study with its increased examination of institutional collections and additional targeted field collecting demonstrated a very large increase (almost 10 times) in apparent endemic species when limited to those only recorded from the state of Oaxaca. Of course, some of this is the result of the fact that many of these species could not be determined to species; therefore, only recorded as Oaxaca, and may either be new to science or previously rarely collected and they may in fact have somewhat broader distribution outside Oaxaca. However, when endemism is extended to the states directly surrounding Oaxaca, a more conservative and probably more realistic demonstration of Oaxacan Alticinae species endemism is revealed of 55%. Of these 41% (113 species) are currently known to be restricted to the state of Oaxaca and 14% (38 species) are known from Oaxaca and the surrounding states.

As mentioned above, one of the objectives of this study is to demonstrate how a variety of research strategies provides a comprehensive account of the diversity for particular region through a combination of researching historical literature, examination of historical collections, and fieldwork. Figure 7 illustrates this on the left-hand histogram through the total results of this study of Oaxacan species and genera of Alticinae. However, to demonstrate the effectiveness of targeted expert fieldwork the middle histogram bars show the 1991 (5 days), 1997 (2 days) and 2010 fieldwork by the author combined and those on the right-hand show the Alticinae diversity captured only for the more extensive (7 day) trip in 2010. The 2010 fieldwork produced 79% of the species and 90% of the genera collected during the author’s fieldwork. However, this may also reveal something about seasonality for collecting Alticinae in Oaxaca; that is, it is best earlier in the season (July rather than August), especially because the majority of collecting in 1991 was in the Sierra de Juárez, like in 2010.

As demonstrated in Figure 9 it is interesting to review the Alticinae taxa of Oaxaca and to note how many species are represented in each genus. For 26 genera (38%) there is only a single species known, whereas there is one genus that has 24 species (9 %) and 82 species (30%) in 4 genera (6%) are represented by single genera. At this time it is not evident the exact cause of this, yet it is still of interest to see this U-shaped curve of species to genera.

Biogeographically it is not surprising that 81% of the genera of Alticinae in Oaxaca show a Neotropical affinity. Other Coleoptera groups also show a strong Neotropical affinity in Mexico overall such as for the species of Curculionidae (41%) (Anderson and O’Brien 1996) and Carabidae at the generic level (40%) are Neotropical (Ball and Shpeley 2000). The geographic position of the state of Oaxaca that includes the extreme “bottleneck” like constriction of the relatively flat Isthmus of Tehuantepec is apparently very important biogeographically and apparently even serves as a kind of transition zone between the more southern Neotropical fauna and the more northern Nearctic fauna. It is probably here that the strong Neotropical influence begins to filter northwards as indicated in Furth (2004) within the southern, more tropical climates of Veracruz and Guerrero. As reported in Furth (2006) the high species diversity in all of Mexico generally is in the southern states of Veracruz (198), Guerrero (141), Oaxaca (122), Chiapas (81), and Tabasco (81). This diversity is certainly influenced strongly by the Neotropical affinities of the taxa. As a result of the current study Oaxaca has jumped to first place among Mexican states as the most Alticinae-diverse, with 275 species – a combination of more extensive examination of collections and the 2010 fieldwork.

In this particular study the 2010 expert fieldwork was done only in one relatively small area of this large tropical state (Oaxaca), i. e., Sierra de Juárez. Given the fact that Oaxaca has many other kinds of habitats and geography (see Maps) one would expect the actual Alticinae diversity to be significantly greater. When other areas of Oaxaca are sampled and even more research collections examined this fact will certainly be realized.

**Figure 15.**
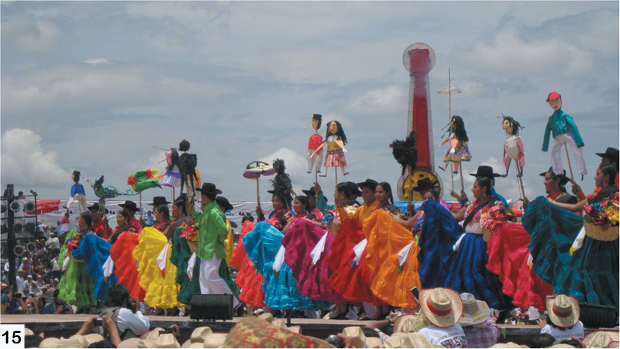
Guelaguetza festival, 2010, in Oaxaca City, performers of the indigenous ethnic group pictured here are from the Pinotepa Nacional people from southern Oaxaca.

The flora and fauna of Oaxaca is truly diverse demonstrated here by the Alticinae, but the people and culture of Oaxaca is also especially diverse and endemic as can be experienced in the annual festival celebrating this cultural diversity – the Guelaguetza (Figure 15).

## Appendix

**Table:** List of genera and species names, species authors for Alticinae currently known from Oaxaca. Also listed in the columns are the known distribution in Mexican states outside of Oaxaca (see list below) for these standard state abbreviations), the sources for any records found by the author in various institutional collections (see Methods for abbreviations), and records from the author’s fieldwork indicated as DGF1991, DGF1997, DGF2010. OM sp.1 indicates a morphospecies name (one that cannot currently be associated with any already described species) found by the author in Oaxaca, Mexico, i.e., OM. Taxon names with a “?” afterwards have some question as to the correct determination of this species. A species with a “?” after the state acronym means that there is some question as to the certainty of the locality from that state. Author names for genera can be found in Furth and Savini (1996, 1998). The references for this Appendix are listed separately.<br/>

**Taxon**	**Distribution**	***Literature source***	***Collections source***	***Author fieldwork***
*Acallepitrix* OM sp. 1			UCB	
*Acanthonycha* OM sp. 1			UCB	
*Acrocyum sallei* Jacoby	OAX	Jacoby 1885		
*Alagoasa acutangula* (Jacoby)	CHIS, COL, DGO, GRO, JAL, MEX, MOR, NL, OAX, VER	Jacoby 1886	MCZ, USNM, UCB	
*Alagoasa bipunctata* (Chevrolat)	CHIS, DF, OAX, SLP, VER, YUC	Jacoby 1886	MCZ, USNM, UCB	
*Alagoasa ceracollis* (Say)	CHIS, DGO, MOR, OAX, VER	Jacoby 1885	USNM	
*Alagoasa chevrolati* (Baly)	OAX, VER, YUC	Furth and Savini 1996	MCZ	
*Alagoasa clypeata* (Jacoby)	CHIS, DGO, HGO, MICH, OAX, TAB, VER	Jacoby 1892	USNM, UCB	
*Alagoasa decemguttatus* (Fabricius)	CHIH, CHIS, COL, DGO, GRO, JAL, MEX, MOR, NAY, OAX, QROO, SON, TAB, VER	Jacoby 1886, Pallister 1953	MCZ, USNM, UCB, UCD, BYU	
*Alagoasa extrema* (Harold)	MOR, OAX, TAB, VER	Jacoby 1886	MCZ, USNM	
*Alagoasa fimbriata* (Forster)	GRO, MICH, MOR, OAX	Jacoby 1886	MCZ, USNM	
*Alagoasa hoegei* (Jacoby)	OAX, VER	Jacoby 1886		
*Alagoasa infirma* (Jacoby)	OAX, VER	Jacoby 1886		
*Alagoasa lateralis* (Jacoby)	COL, GRO, JAL, MEX, MICH, MOR, NAY, OAX	Jacoby 1886	MCZ, USNM	
*Alagoasa longicollis* (Jacoby)	OAX	Jacoby 1886		
*Alagoasa seriata* (Baly)	GRO, MOR, OAX, PUE, VER	Jacoby 1886	MCZ, NHMB, UCB	
*Alagoasa tehuacana* Bechyné	JAL, PUE	Bechyné 1955	USNM, BYU, CAS, UCB	
*Alagoasa violaceomarginata* (Jacoby)	OAX	Jacoby 1886		
*Alagoasa virgata* (Harold)	CHIH, CHIS, COL, DGO, GRO, JAL, MEX, MOR, NAY, PUE, OAX, SIN, SLP, TAB, VER	Jacoby 1886, Pallister 1953	MCZ, USNM, NHMB	
*Alagoasa* OM sp. 1			UCB	
*Alagoasa* OM sp. 2			UCB, UCD	
*Alagoasa* OM sp. 3			UCB	
*Allochroma balyi* Clark	OAX	Jacoby 1886	BYU	
*Allochroma godmani* Jacoby	OAX, VER	Jacoby 1886		
*Allochroma hoegei* Jacoby	DGO, OAX, VER	Jacoby 1886	MCZ	
*Allochroma semipunctatum* Jacoby	OAX	Jacoby 1886		
*Allochroma* OM sp. 1			BYU	
*Altica bimarginata* (Say)	DGO, GRO, OAX, VER	Jacoby 1884	MCZ	
*Altica patruelis* Harold	DF, GRO, GTO, MEX, MICH, OAX ?, PUE, TAB, VER	Jacoby 1884	MCZ, USNM	
*Altica rugicollis* Jacoby	CHIH, OAX	Jacoby 1884	MCZ	
*Altica* OM sp. 1			UCB	
*Asphaera abdominalis* (Chevrolat)	AGS, CHIH, CHIS, COAH, COL, DF, DGO, GRO, GTO, HGO, JAL, MEX, MICH, MOR, NL, OAX, SIN, SLP, TAMPS, VER, ZAC	Jacoby 1885, Jacoby 1892, Pallister 1953	MCZ, USNM, CAS, UCB	
*Asphaera abdominalis var*.			UCB	
*Asphaera cyanopsis* Harold	DF, DGO, OAX, SLP, TAB, VER	Jacoby 1885	MCZ, USNM	
*Asphaera icteridera* (Harold)	CHIS, DGO, GRO, MOR, OAX, VER	Jacoby 1885, Pallister 1953	USNM	DGF 2010
*Asphaera mexicana* (Harold)	CHIS, DGO, GRO, MICH, MOR, NAY, OAX, VER	Jacoby 1886	MCZ, USNM, UCB, UCD	
*Asphaera polita* Jacoby	OAX, TAB, VER	Jacoby 1885	MCZ	
*Asphaera reichei* (Harold)	CHIS, DF, OAX, SLP, VER	Jacoby 1885	USNM	
*Asphaera* OM sp. 1			AMNH	
*Asphaera* OM sp. 2			USNM	
*Blepharida bryanti* Furth	CHIS, OAX	Furth 1998		
*Blepharida flavocostata* Jacoby	GRO, MEX, MICH, MOR, OAX, PUE	Furth 1998		
*Blepharida godmani* Jacoby	CHIS, OAX, VER	Furth 1998		
*Blepharida melanoptera* (Fall)	MICH, OAX, SON	Furth 1998		
*Blepharida mexicana* Jacoby	OAX, VER	Furth 1998		
*Blepharida punctatissima* Jacoby	CHIS, OAX, VER	Furth 1998		
*Blepharida quatuordecimpunctata* Jacoby	CHIS, OAX, VER	Furth 1998		
*Blepharida rhois* (Forster)	CHIH, COAH, DGO, GRO, HGO, NL, OAX, PUE, QRO, SLP, TAMPS	Furth 1998		
*Blepharida trifasciata* Jacoby	OAX	Furth 1998		
*Blepharida unami* Furth	OAX, PUE	Furth 1998		
*Blepharida verdea* Furth	GRO, MOR, OAX	Furth 1998		
*Cacoscelis flava* Clark	OAX, TAMPS	Jacoby 1884	USNM	
*Cacoscelis varians* (Jacoby)	OAX, TAB, VER	Jacoby 1891		
*Capraita conspurcata* (Jacoby)	CHIS, DF, DGO, GRO, GTO, HGO, MEX, MICH, MOR, OAX, PUE, VER	Jacoby 1886	MCZ, USNM	DGF 2010
*Capraita maculata* (Harold)	CHIS, GRO, JAL, MEX, MOR, OAX, VER, YUC	Jacoby 1886	MCZ	
*Centralaphthona fulvipennis* ? Jacoby	VER ?		MCZ, UCB	
*Centralaphthona mexicana* Jacoby	COAH, DGO, GRO	Jacoby 1885, Jacoby 1891	MCZ: BYU	
*Centralaphthona obscuripennis* (Jacoby)	GRO, MOR		USNM, CAS	DGF 1991, DGF 2010
*Centralaphthona semipuncata* Jacoby	JAL, VER	Jacoby 1891	MCZ, UCB	DGF 1991
*Chaetocnema balyi* Jacoby	COAH, DF	Jacoby 1892	MCZ, CAS, CDFA, BYU, UCB	
*Chaetocnema capitata* Jacoby	DGO, GTO	Jacoby 1885	MCZ	DGF 2010, DGF 1991
*Chaetocnema cephalotes* Jacoby	PUE, SIN		NHMB, AMNH, UCB	
*Chaetocnema confinis* Crotch	DF		USNM, BYU, CDFA	
*Chaetocnema fulvicornis* Jacoby	DGO, GRO, GTO	Jacoby 1885		DGF 2010
*Chaetocnema fulvilabris* Jacoby	GRO, MOR, VER	Jacoby 1892	UCB, USNM	
*Chaetocnema minuta* Melsheimer			CAS, UCB	DGF 2010
*Chaetocnema* OM sp. 1				DGF 2010
*Chaetocnema* OM sp. 2			BYU	
*Chaetocnema* OM sp. 3				DGF 2010
*Chaetocnema* OM sp. 4			BYU	
*Chrysogramma septempunctata* Jacoby	DGO, MOR, OAX, PUE	Furth and Savini 1996	USNM	
*Chrysogramma trifasciata* Jacoby	OAX	Jacoby 1891		
*Cyrsylus recticollis* Jacoby	CHIS, TAB, VER	Jacoby 1892	USNM, TAMU	
*Deuteraltica longicornis* (Jacoby)	CHIS		USNM, TAMU	
*Deuteraltica* OM sp. 1			TAMU	
*Dibolia championi* Jacoby	OAX, VER	Parry 1974	USNM	
*Dinaltica* OM sp. 1			BYU	
*Dinaltica* OM sp. 2			USNM	
*Dinaltica* OM sp. 3			BYU	DGF 2010
*Dinaltica* OM sp. 4				DGF 2010
*Diphaltica nitida* (Jacoby)	CHIS, DF, DGO, MICH, OAX, TAB, VER	Jacoby 1884	MCZ, USNM	
*Diphaltica* OM sp. 1			CAS	
*Diphaulaca aulica* (Olivier)			UCB	DGF 1997
*Diphaulaca aulica cordobae* Barber	CHIS, GRO, GTO, HGO, JAL, MEX, MICH, MOR, NAY, OAX, PUE, QROO, SLP?, TAB, TAMPS, VER, YUC	Jacoby 1884	MCZ, USNM, BYU, UCB	
*Diphaulaca wagneri* Harold	CHIS, GRO, OAX, YUC	Barber 1941	NHMB	DGF 2010
*Disonycha discoidea abbreviata* Melsheimer	DGO, MEX, MOR, OAX	Jacoby 1884	MCZ	
*Disonycha antennata* Jacoby	COL, DGO, GRO, JAL, MEX, MICH, MOR, OAX, VER	Jacoby 1884, Blake 1955	USNM	
*Disonycha brevilineata* Jacoby	DGO, GRO, JAL, MOR, OAX	Jacoby 1884, Jacoby 1902, Blake 1955	MCZ, CAS	
*Disonycha brunneofasciata* Jacoby	GRO, PUE, SLP	Blake 1955	USNM, UCB	
*Disonycha caroliniana* (Fabricius)	DGO, NL, OAX, SIN, VER	Jacoby 1884	USNM	
*Disonycha collata*<br/>(Fabricius)	CHIH, COAH, DF, DGO, GTO, JAL, MEX, MICH, MOR, OAX, PUE, TAB, VER, YUC	Jacoby 1884, Pallister 1953	MCZ, USNM	
*Disonycha dorsata* Harold	MOR, OAX, TAB, VER, YUC	Jacoby 1884	MCZ, USNM, BYU	
*Disonycha figurata* Jacoby	AGS, CHIH, CHIS, COAH, COL, DF, DGO, GRO, GTO, JAL, MEX, MICH, MOR, NAY, OAX, SIN, TAB, VER, YUC	Jacoby 1884, Pallister 1953, Blake 1955	MCZ, USNM, NHMB, UCB	
*Disonycha fumata fumata* LeConte	BC, CHIH, CHIS, DGO, GRO, HGO, JAL, MEX, MICH, MOR, NL, OAX, PUE, SLP, SON, TAB, VER, ZAC	Blake 1955	USNM	
*Disonycha glabrata* (Fabricius)	BC, BCS, CAMP, CHIS, COL, DGO, GRO, JAL, MOR, NAY, OAX, PUE, SIN, SON, TAB, TAMPS, YUC, VER	Jacoby 1884, Blake 1955	MCZ, USNM, CAS, UCB, UCD	DGF 2010
*Disonycha guatemalensis* Jacoby	CHIS, GRO, MOR, OAX, VER?	Blake 1955	USNM	DGF 2010
*Disonycha hoegei* Jacoby	VER, OAX	Jacoby 1884		
*Disonycha leptolineata texana* Schaeffer	DGO, GRO, JAL, MOR, NL, OAX, QROO, TAMPS, YUC	Blake 1955	USNM, CAS	
*Disonycha maculipes* Jacoby	CHIS, VER	Jacoby 1891	USNM, AMNH, CAS, UCB	
*Disonycha militaris* Jacoby	TAB, VER, YUC	Jacoby 1884	USNM, UCB	
*Disonycha nigrita* Jacoby			UCB	DGF 2010
*Disonycha pluriligata* LeConte	BC, CHIH, DGO, JAL, NAY, SIN, SLP, SON, VER	Furth and Savini 1996	MCZ, UCB	
*Disonycha politula* Horn	AGS, CAMP, CHIH, DF, DGO, GRO, GTO, HGO, JAL, MEX, MOR, OAX, PUE, QRO, SLP, SON, TAMPS, VER, ZAC	Jacoby 1891, Pallister 1953	MCZ, USNM	
*Disonycha quinquelineata* (Latreille)	CHIS, COL, GRO, OAX, QROO, TAB, TAMPS, VER	Jacoby 1884, Blake 1955	MCZ, USNM	
*Disonycha scriptipennis* (Jacoby)	CHIS, COL, DGO, GRO, MOR, NAY, OAX, YUC	Jacoby 1891	USNM, NHMB	
*Disonycha subaenea* Jacoby	DGO, GRO, MOR, OAX	Jacoby 1884	MCZ, USNM	
*Disonycha teapensis* Blake	OAX, SLP, TAB	Blake 1955	NHMB	
*Disonycha* OM sp. 1			CDFA	
*Distigmoptera suturalis* (Jacoby)	GRO, OAX	Jacoby 1892	NHMB	
*Dysphenges* OM sp. 1				DGF 2010
*Egleraltica* OM sp. 1			BYU, UCB	
*Epitrix cucumeris* (Harris)	DGO, GRO, MOR, PUE, VER	Jacoby 1891	MCZ, USNM, ZSMC, CDFA, UCB	DGF 1991, DGF 2010
*Epitrix fasciata* Blatchley	CHIH, DGO, NL, TAMPS	Maes and Staines 1991	USNM	DGF 2010
*Epitrix robusta* Jacoby	GRO	Jacoby 1891		DGF 2010, DGF 1997
*Epitrix rufula* Weise	DF, GRO, MOR	Jacoby 1891	USNM, UCB	DGF 2010
*Epitrix* OM sp.1			UCB	
*Epitrix* OM sp.2				DGF 2010
*Epitrix* OM sp.3			CDFA	DGF 2010
*Epitrix* OM sp.4				DGF 2010
*Genaphthona transversicollis* (Jacoby)	CHIS, JAL, OAX, PUE		USNM, BYU, CDFA, UCB	DGF 1997
*Glenidion flexicaulis* Schaeffer	TAMPS, YUC		USNM, TAMU	
*Glyptina nivialis* Horn	MOR		USNM	DGF 2010
*Heikertingerella* OM sp. 1				DGF 2010
*Heikertingerella* OM sp. 2			BYU	
*Heikertingerella* OM sp. 3			UCB	
*Heikertingerella* OM sp. 4			UCB	
*Hemiphyrnus elongatus* Jacoby	OAX, TLAX, VER	Jacoby 1884		
*Hemiphyrnus sulcatipennis* (Jacoby)	GRO, MEX, OAX	Jacoby 1891	NHMB, BMNH	
*Hemiphyrnus sydneyae* Gilbert & Andrews			BYU	
*Hemiphyrnus tenuicornis* Jacoby	HGO, OAX	Jacoby 1891	MCZ	
*Hypolampsis* OM sp. 1			UCB	
*Hypolampsis* OM sp. 2			BYU, USNM	
*Hypolampsis* OM sp. 3			BYU	
*Hypolampsis* OM sp. 4			BYU	
*Hypolampsis* OM sp. 5			USNM	
*Iphitroides nigrocinctus* Jacoby	GRO	Jacoby 1891	CAS	
*Kuschelina laeta* (Perbosc)	TAMPS, VER	Heikertinger and Csiki 1940	MCZ, USNM, UCB	
*Kuschelina modesta* (Jacoby)	CHIH, CHIS, DF, DGO, GRO, GTO, HGO, MEX, MOR, OAX, PUE, SLP, TLAX, VER	Jacoby 1886, Pallister 1953	MCZ, USNM	DGF 2010
*Leptophysa hirtipennis* (Jacoby)	OAX, VER		USNM	
*Longitarsus columbicus* ? Harold	GRO		MCZ	DGF 2010
*Longitarsus mexicanus* Csiki	DF, DGO, GRO, GTO, HGO, MEX, MICH, MOR, PUE	Jacoby 1891	MCZ, NHMB, USNM, UCB	DGF 1997
*Longitarsus varicornis* Suffrian	TAB, VER	Jacoby 1885, Jacoby 1891	UCB	
*Longitarsus* OM sp. 1			BYU	
*Longitarsus* OM sp. 2			UCB	DGF 1997
*Longitarsus* OM sp. 3			CAS	
*Longitarsus* OM sp. 4			UCB	
*Longitarsus* OM sp. 5			UCB	
*Longitarsus* OM sp. 6			USNM	
*Luperaltica longicornis* (Jacoby)	CHIS, COL?, MOR?, OAX?		USNM	
*Luperaltica sylvia* (Bechyne & Bechyne)			USNM, UCB	DGF 1997, DGF 2010
*Luperaltica viridipennis* (Jacoby)	OAX	Jacoby 1884		
*Luperaltica* OM sp. 1			USNM	
*Luperaltica* OM sp. 2			BYU	
*Luperaltica* OM sp. 3			BYU, USNM	
*Luperaltica* OM sp. 4			BYU	
*Lupraea frontalis* (Jacoby)	OAX	Jacoby 1885	USNM	
*Lupraea fulvicollis* ? Jacoby	VER		MCZ	DGF 2010
*Lupraea guatemalensis* (Jacoby)	CHIS, GRO, MOR, VER	Jacoby 1891	BYU, CDFA, USNM, UCB	
*Lupraea semifulva* (Jacoby)	CHIS, OAX		USNM	
*Lupraea smithi* (Jacoby)	GRO, MOR	Jacoby 1891	USNM	DGF 2010
*Lupraea* OM sp. 1			BYU	
*Lupraea* OM sp. 2			UCB	
*Lupraea* OM sp. 3			UCD	
*Lysathia jacobyi* (Csiki)	DF, GTO, OAX, TAB, ZAC	Jacoby 1891	USNM	
*Lysathia occidentalis* (Suffrian)	YUC		ZSMC, UCB	
*Macrohaltica patruelis* (Harold)	DF, DGO, GTO, MEX, MICH, MOR, OAX, PUE, VER	Jacoby 1884	USNM	
*Macrohaltica* OM sp. 1			UCB	
*Margaridisa managua* ? (Bechyné)	DGO, SLP		USNM	DGF 2010
*Monomacra cupreata* (Jacoby)	OAX	Jacoby 1891		
*Monomacra hoegei* (Jacoby)	OAX, VER	Jacoby 1884		
*Monomacra mexicana* (Jacoby)	OAX, VER	Jacoby 1884		
*Monomacra tibialis* (Olivier)	OAX		USNM	
*Monomacra violacea* (Jacoby)	CHIS, VER		USNM, BYU, CAS, UCB	DGF 2010
*Monomacra* OM sp. 1			UCB	
*Monomacra* OM sp. 2			USNM	
*Neothona sp*.	JAL, MICH, OAX, VER		USNM	
*Neothona* OM sp. 1				DGF 2010
*Neothona* OM sp. 2			USNM, UCB	
*Nesaecrepida infuscata* (Schaeffer)	CAMP, COL, GRO, JAL, MICH, OAX, TAB, TAMPS, VER		USNM, ZSMC, UCB	
*Notozona histrionica* Baly	OAX, VER	Furth and Savini 1996	MCZ	
*Omophoita aequinoctialis aequinoctialis* (Linnaeus)	CHIS, HGO, MICH, OAX, QROO, SLP, TAB, TAMPS, VER	Furth and Savini 1996	USNM, AMNH, BMNH, UCB, UCD	
*Omophoita cinctipennis* (Chevrolat)	JAL, OAX, PUE, SLP, VER	Jacoby 1885	USNM	
*Omophoita octomaculata* (Crotch)	OAX, TAB, TAMPS, VER	Jacoby 1886	ZSMC	
*Omophoita quadrinotata centraliamericana* Bechyné	OAX, TAB, VER	Bechyné 1955	USNM, BMNH	
*Omophoita recticollis* (Baly)	CHIS, HGO, OAX, TAB, TAMPS, VER	Jacoby 1885, 1891	USNM	
*Palaeothona chiriquiensis* Jacoby				DGF 2010
*Palaeothona rubroviridis* Blake	DGO	Blake 1950		DGF 2010
*Palaeothona rugifrons* (Jacoby)	VER	Jacoby 1885	BYU, UCB, USNM	
*Palaeothona* OM sp. 1			CDFA	
*Palaeothona* OM sp. 2			USNM	
*Palaeothona* OM sp. 3			BYU	
*Palaeothona* OM sp. 4			BYU	
*Palaeothona* OM sp. 5			UCB	
*Palaeothona* OM sp. 6			BYU, CDFA	
*Pedilia inornata* (Jacoby)	OAX, VER	Duckett 1993 in litt.		
*Phrynocepha capitata* Jacoby	CHIS?, GRO, JAL, OAX, TAB?	Jacoby 1884	USNM, UCB	
*Phrynocepha deyrollei* Baly	AGS, CHIH, DGO, GRO, GTO, MICH, MOR, OAX, PUE, SLP ?	Jacoby 1884, Pallister 1953	USNM, UCB	DGF 2010
*Phrynocepha pulchella* Baly	CHIS, COL, DGO, GTO, JAL, MICH, MOR, OAX, VER	Jacoby 1884	USNM, NHMB	
*Phydanis bicolor* Horn	OAX, TAMPS		USNM	
*Phydanis nigriventris* Jacoby	GRO, OAX, SLP, SON	Jacoby 1891	USNM	DGF 2010
*Phyllotreta aeneicollis* Crotch				DGF 1997, DGF 2010
*Phyllotreta pusilla* Horn	AGS, BC?, CHIH, DF, DGO, HGO, MOR, OAX, ZAC	Chittenden 1923	USNM	DGF 1997
*Phyllotreta* OM sp. 1				DGF 2010
*Phyllotreta* OM sp. 2			UCB	
*Physimerus scabrosus* (Clark)	DGO, OAX, VER	Jacoby 1886	MCZ	
*Physimerus* OM sp. 1			CAS	
*Platiprosopus pallens* (Fabricius)	GRO, HGO, MOR, OAX, PUE, VER	Furth and Savini 1996	USNM	
*Plectrotetra clarki* Baly	DF, DGO, HGO, MOR, OAX, PUE, SIN, SLP, TAMPS, VER	Jacoby 1884	MCZ, USNM	
*Plectrotetra guatemalensis* Jacoby	MOR	Jacoby 1891	BMNH	
*Plectrotetra inaequalis* Jacoby	OAX, TAMPS, VER	Jacoby 1884	USNM	
*Plectrotetra multipunctata* Jacoby	DGO?, MEX, MOR, OAX, VER	Jacoby 1891	MCZ, USNM	
*Plectrotetra submetallica* Jacoby	OAX, VER	Jacoby 1884		
*Prasona viridis* Baly	VER	Jacoby 1886	USNM	
*Prasona* OM sp. 1			BYU, USNM	
*Propiasus fulvus* (Jacoby)	GRO	Jacoby 1892	USNM	
*Pseudorthygia nigritarsis* Jacoby	GRO, OAX, TAMPS	Jacoby 1891	USNM	DGF 2010
*Psylliodes convexior* LeConte	BCS	Horn 1895, Furth and Savini 1998		DGF 1997
*Resistenciana ornata* (Jacoby)	PUE, VER	Jacoby 1884	MCZ, BYU, USNM	
*Rhinotmetus modestus* Jacoby	GRO, MOR	Jacoby 1892	MCZ	DGF 1991
*Rhinotmetus* OM sp. 1			BYU	
*Rhinotmetus* OM sp. 2				DGF 1991
*Rhinotmetus* OM sp. 3				DGF 1991
*Scelidopsis rufofemorata* Jacoby	TAMPS, VER	Jacoby 1888	USNM, CAS	
*Sphaeronychus* OM sp. 1			BYU	
*Sphaeronychus* OM sp. 2			BYU, UCB, USNM	
*Stegnea* OM sp. 1			TAMU	
*Strabala rotunda* Blake	CHIS, COL, DF, GRO, JAL, NAY, NL, SLP, TAMPS, VER, YUC	Blake 1953	USNM, NHMB, ZSMC, UCB	
*Strabala rufa* Illiger	CHIS, COL, DGO, GRO, OAX, PUE, TAB, VER	Jacoby 1884, 1891		
*Syphrea burgessi* (Crotch)	MOR, OAX, TAMPS		USNM	
*Syphrea cyaneipennis* (Jacoby)	GRO, HGO, JAL, SLP, TAB, TAMPS	Jacoby 1891	USNM, BYU, CDFA	
*Syphrea flavicollis* (Jacoby)	BCS, GRO, GTO, JAL, MOR, OAX, PUE	Jacoby 1884, Riley, Clark and Gilbert 2001	MCZ, USNM	
*Syphrea parvula* (Jacoby)	JAL, TAB, VER, YUC	Jacoby 1891	USNM, BYU, UCB	
*Syphrea smithi* (Jacoby)	OAX, TAB, TAMPS	Jacoby 1891	USNM	
*Syphrea sublaevipennis* (Jacoby)	OAX, VER	Jacoby 1891	MCZ	
*Syphrea teapensis* (Jacoby)	OAX, SLP, TAB, VER	Jacoby 1891	USNM	
*Syphrea* OM sp. 1			BYU	DGF 2010
*Syphrea* OM sp. 2			BYU	
*Syphrea* OM sp. 3			CAS	
*Syphrea* OM sp. 4			BYU	
*Syphrea* OM sp. 5			BYU	
*Syphrea* OM sp. 6				DGF 2010
*Syphrea* OM sp. 7			USNM	
*Syphrea* OM sp. 8			BYU	
*Syphrea* OM sp. 9			TAMU, USNM	
*Systena abbreviata* Jacoby	PUE	Jacoby 1902	CDFA	
*Systena blanda* Melsheimer	BC?, CHIH, JAL, MICH, NL, SIN, SLP?, SON, TAB, VER	Pallister 1953	USNM, UCB	
*Systena championi* Jacoby	GRO, MOR, OAX, VER		USNM	
*Systena contigua* Jacoby	CHIS, GRO, GTO, HGO, OAX, SON?, TAMPS, VER?, ZAC	Jacoby 1884	USNM, CDFA, UCB	DGF 2010
*Systena gracilenta* Blake	NL	Blake 1933, Furth and Savini 1998		DGF 2010
*Systena nigroplagiata* Jacoby	AGS, CHIH, DF, DGO, GTO, GRO, JAL, MICH, MOR, OAX, PUE, VER	Jacoby 1884, Pallister 1953	MCZ, USNM	DGF 2010
*Systena pectoralis* Clark	CHIS, GTO, OAX, VER	Jacoby 1884	MCZ	
*Systena puncticollis* Jacoby	OAX	Jacoby 1884		
*Systena s-littera* (Linnaeus)	CHIS, GTO, TAB, VER	Jacoby 1884	MCZ, USNM, UCB	
*Systena semivittata* Jacoby	BCS, GRO, GTO, HGO, MEX, MOR, NL, OAX, SIN	Jacoby 1884	MCZ, USNM, NHMB	DGF 2010
*Systena subcostata* Jacoby	MICH, MOR, VER	Jacoby 1884	USNM, CDFA, UCB	DGF 2010
*Systena sulphurea* Jacoby	CHIH, DGO, GRO, MOR, OAX	Jacoby 1891	MCZ, USNM, BYU	DGF 1997
*Systena thoracica* Jacoby	CAMP, HGO, PUE, QROO, TAB, VER	Jacoby 1884	MCZ, USNM, UCB	
*Systena variabilis* Jacoby	CHIH, CHIS, COL, DGO, GRO, GTO, MICH, MOR, NAY, OAX, VER	Jacoby 1884, Pallister 1953	MCZ, USNM, ZSMC, UCB	
*Systena* OM sp. 1			UCB	DGF 2010
*Systena* OM sp. 2				DGF 2010
*Systena* OM sp. 3			UCB	
*Systena* OM sp. 4			UCB	
*Systena* OM sp. 5			UCB	
*Systena* OM sp. 6			UCB, USNM	
*Systena* OM sp. 7			UCB	
*Systena* OM sp. 8			UCB	
*Systena* OM sp. 9			BYU	
*Systena* OM sp. 10			UCB	
*Trichaltica zapotensis* (Jacoby)			BYU, CDFA, TAMU, UCB, USNM	
*Trichaltica* OM sp. 1			CDFA, TAMU	DGF 2010
*Walterianella inscripta* (Jacoby)	OAX, SLP, VER	Jacoby 1886	MCZ, USNM	
*Walterianella sublineata* (Jacoby)	OAX, TAB, VER, YUC	Jacoby 1886	MCZ, USNM, UCD	
*Walterianella* OM sp. 1			UCB	
New Genus A ? OM sp. 1			USNM	
New Genus B ? OM sp. 1			UCB	

Mexican States (Abbreviations): Aguascalientes (AGS); Baja California (BC); Baja California Sur (BCS); Campeche (CAMP); Chiapas (CHIS); Chihuahua (CHIH); Coahuila (COAH); Colima (COL); Distrito Federal (DF); Durango (DGO); Guanahuato (GTO); Guerrero (GRO); Hidalgo (HGO); Jalisco (JAL); Mexico (MEX); Michoacan (MICH); Morelos (MOR); Nayarit (NAY); Nuevo Leon (NL); Oaxaca (OAX); Puebla (PUE); Queretaro (QRO); Quintana Roo (QROO); San Luis Potosi (SLP); Sinaloa (SIN); Sonora (SON); Tabasco (TAB); Tamaulipas (TAMPS); Tlaxcala (TLAX); Veracruz (VER); Yucatan (YUC); Zacatecas (ZAC).
